# Correction to “Representation of Differential Learning Method for Mitosis Detection”

**DOI:** 10.1155/johe/9861404

**Published:** 2026-04-26

H. Ali, H. Li, E. A. Retta, et al., “Representation of Differential Learning Method for Mitosis Detection,” *Journal of Healthcare Engineering*, 2021, 6688477, https://doi.org/10.1155/2021/6688477.

In the article, similarities were identified in the images shown in Figure 1. Specifically:

• In Figure 1(b), the 3rd cell in the 1st row and the 8th cell in the 2nd row are similar.
• In Figure 1(b), the 5th cell in the 1st row and 6th cell in the 2nd row are similar.
• In Figure 1(a), the 6th cell in the 1st row is similar to the 5th and 6th cells in the 1st and 2nd rows of Figure 1(b).

The authors have provided a correct Figure 1 as below, and the editorial board has confirmed the results and conclusions presented are unaffected by the errors.

FIGURE 1(a) Blue rectangle represents the actual mitotic cells, and (b) red rectangle represents the nonmitotic cells, which have a similar morphological appearance.

**Figure:**
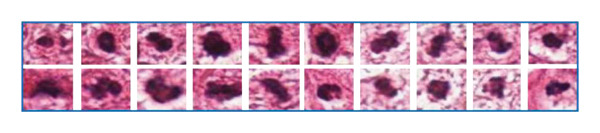
(a)

**Figure:**
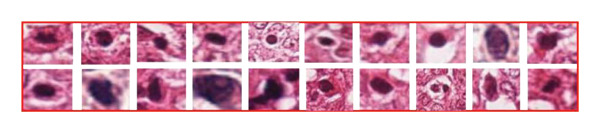
(b)

We apologize for these errors.

